# Supplementary material for: Hot spot prediction in protein-protein interactions by an ensemble system
Source: BMC Syst Biol. 2018 Dec 31;12(Suppl 9):132. doi: 10.1186/s12918-018-0665-8 (PMC6311905; doi:10.1186/s12918-018-0665-8)
Supplement: Supplementary file 2 — All relevant MATLAB code. A simple Matlab implement of our predictor. (ZIP 19,742 kb) [file 12918_2018_665_MOESM2_ESM.zip › Additional file 2/Read ASA/ASA website/dbMPIKT/2MVMA.html]

 NetSurfP - Protein Surface Accessibility and Secondary Structure Predictions

  

|  |  |  |
| --- | --- | --- |
|  |  | NetSurfP - Protein Surface Accessibility and Secondary Structure Predictions  Technical University of Denmark |

  


---

```
# For publication of results, please cite:
# A generic method for assignment of reliability scores applied to solvent accessibility predictions.
# Bent Petersen, Thomas Nordahl Petersen, Pernille Andersen, Morten Nielsen and Claus Lundegaard
# BMC Structural Biology 2009, 9:51 doi:10.1186/1472-6807-9-51
#
# Column 1: Class assignment - B for buried or E for Exposed - Threshold: 25% exposure, but not based on RSA
# Column 2: Amino acid
# Column 3: Sequence name
# Column 4: Amino acid number
# Column 5: Relative Surface Accessibility - RSA
# Column 6: Absolute Surface Accessibility
# Column 7: Z-fit score for RSA prediction
# Column 8: Probability for Alpha-Helix
# Column 9: Probability for Beta-strand
# Column 10: Probability for Coil
E G  2MVMA                  1    0.876  68.925  -0.857   0.003   0.003   0.994
E P  2MVMA                  2    0.634  89.936  -0.129   0.018   0.019   0.964
E G  2MVMA                  3    0.711  55.964  -0.563   0.018   0.019   0.964
E S  2MVMA                  4    0.555  65.034  -0.711   0.053   0.043   0.903
E E  2MVMA                  5    0.618 108.000  -0.198   0.113   0.043   0.844
E D  2MVMA                  6    0.430  61.905  -0.878   0.184   0.043   0.773
E D  2MVMA                  7    0.499  71.935  -0.649   0.184   0.043   0.773
E D  2MVMA                  8    0.346  49.902  -0.077   0.278   0.093   0.628
B I  2MVMA                  9    0.121  22.348   0.816   0.199   0.152   0.649
E D  2MVMA                 10    0.355  51.127   0.045   0.199   0.152   0.649
B L  2MVMA                 11    0.123  22.485   0.436   0.125   0.227   0.648
B F  2MVMA                 12    0.141  28.359   0.182   0.125   0.227   0.648
B G  2MVMA                 13    0.260  20.478  -1.610   0.018   0.088   0.893
B S  2MVMA                 14    0.348  40.844  -1.014   0.018   0.047   0.935
E D  2MVMA                 15    0.560  80.667  -0.928   0.018   0.019   0.964
E N  2MVMA                 16    0.488  71.370  -0.433   0.115   0.016   0.868
E E  2MVMA                 17    0.617 107.772  -0.520   0.339   0.016   0.645
E E  2MVMA                 18    0.648 113.171  -0.495   0.430   0.016   0.555
E E  2MVMA                 19    0.408  71.278   0.044   0.430   0.016   0.555
E D  2MVMA                 20    0.394  56.718  -0.069   0.502   0.002   0.495
E K  2MVMA                 21    0.684 140.802   0.879   0.858   0.002   0.139
E E  2MVMA                 22    0.479  83.681   0.456   0.923   0.002   0.076
B A  2MVMA                 23    0.098  10.822  -0.874   0.923   0.002   0.076
E A  2MVMA                 24    0.436  48.036   0.534   0.923   0.002   0.076
E Q  2MVMA                 25    0.509  90.961   1.459   0.923   0.002   0.076
B L  2MVMA                 26    0.086  15.765   0.769   0.923   0.002   0.076
B R  2MVMA                 27    0.256  58.670   1.009   0.923   0.002   0.076
E E  2MVMA                 28    0.455  79.419   0.276   0.970   0.001   0.030
E E  2MVMA                 29    0.553  96.574   0.944   0.970   0.001   0.030
B R  2MVMA                 30    0.225  51.571   0.848   0.970   0.001   0.030
B L  2MVMA                 31    0.073  13.458   0.009   0.970   0.001   0.030
E R  2MVMA                 32    0.501 114.706   1.284   0.970   0.001   0.030
E Q  2MVMA                 33    0.514  91.854   1.027   0.970   0.001   0.030
B Y  2MVMA                 34    0.138  29.491   0.854   0.923   0.002   0.076
B A  2MVMA                 35    0.237  26.172  -0.758   0.923   0.002   0.076
E E  2MVMA                 36    0.665 116.088   0.440   0.858   0.002   0.139
E K  2MVMA                 37    0.523 107.519   0.462   0.782   0.003   0.216
E K  2MVMA                 38    0.482  99.189   0.124   0.600   0.003   0.397
E A  2MVMA                 39    0.415  45.678  -0.468   0.406   0.004   0.590
E K  2MVMA                 40    0.645 132.738   0.550   0.176   0.004   0.820
E K  2MVMA                 41    0.512 105.277   0.399   0.053   0.005   0.942
E P  2MVMA                 42    0.471  66.778  -1.356   0.016   0.005   0.979
E A  2MVMA                 43    0.637  70.241  -1.793   0.018   0.019   0.964
E L  2MVMA                 44    0.658 120.571  -1.490   0.003   0.003   0.994

Explain the output.  Go back.
```

---
